# Infrared Cooling in an Anharmonic Cascade Framework: 2‑Cyanoindene, the Smallest Cyano-PAH Identified in Taurus Molecular Cloud‑1

**DOI:** 10.1021/acsearthspacechem.4c00381

**Published:** 2025-02-04

**Authors:** Mark H. Stockett, Vincent J. Esposito, Eleanor K. Ashworth, Ugo Jacovella, James N. Bull

**Affiliations:** † Department of Physics, 7675 Stockholm University, SE-10691 Stockholm, Sweden; ‡ 53406 NASA Ames Research Center, Moffett Field, California 94035, United States; § Chemistry, Faculty of Science, 6106 University of East Anglia, Norwich NR4 7TJ, United Kingdom; ∥ 329018 Institut des Sciences Moléculaires d’Orsay, Centre National de la Recherche Scientifique (CNRS), Université Paris-Saclay, F-91405 Orsay, France

**Keywords:** astrochemistry, vibrational spectroscopy, emission rates, aromatic infrared bands, anharmonicity, polycyclic aromatic hydrocarbon, radiative stabilization

## Abstract

Infrared (IR) cooling of polycyclic aromatic hydrocarbon (PAH) molecules is a major radiative stabilization mechanism of PAHs present in space and is the origin of the aromatic infrared bands (AIBs). Here, we report an anharmonic cascade model in a master equation framework to model IR emission rates and emission spectra of energized PAHs as a function of internal energy. The underlying (simple harmonic cascade) framework for fundamental vibrations has been developed through the modeling of cooling rates of PAH cations and other carboneaous ions measured in electrostatic ion storage ring experiments performed under “molecular cloud in a box” conditions. The anharmonic extension is necessitated because cyano-PAHs, recently identified in Taurus Molecular Cloud-1 (TMC-1), exhibit strong anharmonic couplings, which make substantial contributions to the IR emission dynamics. We report an experimental mid-IR (650–3200 cm^–1^) absorption spectrum of 2-cyanoindene (2CNI), which is the smallest cyano-PAH that has been identified in TMC-1 and model its IR cooling rates and emission properties. The mid-IR absorption spectrum is reasonably described by anharmonic calculations at the B3LYP/N07D level of theory that include resonance polyad matrices, although the CN-stretch mode frequency continues to be difficult to describe. The anharmonic cascade framework can be readily applied to other neutral or charged PAHs and is also readily extended to include competing processes, such as recurrent fluorescence and isomerization.

## Graphical abstract

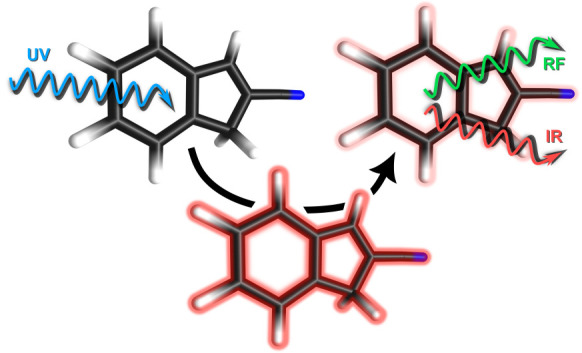

## 1 Introduction

The preponderance of polycyclic aromatic hydrocarbons (PAHs) as a class of molecules in space is highlighted by widespread observations of the aromatic infrared bands (AIBs),^1,2^ which are a set of broad emission bands situated on plateaus with pronounced features at 3030 cm^–1^ (3.3 μm, C–H stretch), 1612 cm^–1^ (6.2 μm, C–C stretch), 1299 cm^–1^ (7.7 μm, C–C stretch), 1163 cm^–1^ (8.6 μm, C–H in-plane bending), and 893 cm^–1^ (11.2 μm, C–H out-of-plane bending).^3^ The AIBs, which are broadly observed by the Spitzer and James Webb space telescopes, are found across most astrochemical environments,^4–6^ with modeling predicting that 10–25% of galactic carbon exists as PAHs.^3,7–9^ The subtle variations in vibrational frequencies with PAH size, isomer, or, in some cases, functional group substitution, means that the AIB spectra cannot be used to identify individual PAHs.^10^ The AIBs are presumed to originate from infrared (IR) cooling (emission) of PAHs following electronic absorption of a visible or near-ultraviolet (UV) photon and internal conversion to the ground electronic state.^11,12^ Lifetimes for internal conversions in isolated PAHs are usually on the picosecond time scale,^13–15^ which is fast compared with lifetimes for direct fluorescence (nanoseconds), followed by intramolecular vibrational energy redistribution also on the picosecond time scale.^16^ In principle, PAHs could also be energized through inelastic collisions with other particles such as atomic ions,^17,18^ which are thought to be the main PAH destruction pathway in astrochemical models.^19^

Over the past few years, a series of specific aromatic and PAHs have been identified in Taurus Molecular Cloud-1 (TMC-1),^19–26^ which is a cold, dark molecular cloud that is considered as the nearest stellar nursery or star-forming region to Earth.^27^ These identifications were made by comparing observed astronomical lines with rotational spectroscopy measurements performed in the laboratory. Some of the specific aromatic molecules and PAHs identified in TMC-1 include benzonitrile,^20^ indene,^21,22^ 2-cyanoindene,^23^ 1-/2-cyanonaphthalene,^19^ 1-/5-cyanoacenaphthylene,^24^ and isomers of cyanopyrene.^25,26^ Interestingly, all of the PAHs were observed in substantially higher abundance, some by several orders of magnitude, than state-of-the-art models predict, implying that astrochemical models overestimate PAH destruction mechanisms and/or underestimate formation and stabilization mechanisms. Although indene and 2-cynanoindene (2CNI) are not true PAHs because of a sp^2^-hybridized carbon in the ring system, they are generally categorized as PAHs in the context of astrochemistry. The majority of the identified PAHs have a cyano (nitrile) functional group, which induces a large dipole moment making them suitable for radioastronomical detection.

In a systematic effort to understand the radiative stabilization mechanisms active in PAHs, several of the current authors have used cryogenic ion storage ring experiments at the DESIREE infrastructure to probe the radiative cooling rates and dynamics of energized PAH cations.^28–31^ A key advantage of the DESIREE infrastructure is that experiments are conducted under so-called “molecular cloud in a box conditions”, where the background temperature (*T* ≈ 13 K) and pressure (*P* ≈ 10^–14^ mbar) are similar to cold, dark molecular clouds.^32^ There, the relaxation dynamics of energized ions can be monitored over the “ultraslow” time scale (milliseconds to seconds) in a collision-free environment.^33^ To support these measurements, we implemented a simple harmonic cascade (SHC) model of vibrational de-excitation based on harmonic oscillators in a master equation framework,^33,34^ which could adequately describe experimental emission rates (by monitoring the disappearance of hot bands or the quenching of dissociation) as a function of total vibrational energy within a factor of 2 or so. Significantly, the DESIREE measurements and others have shown that, in addition to IR cooling, recurrent fluorescence (RF) may occur from thermally populated electronics states.^29,35–37^ On the other hand, the dissociation and radiative cooling rates of some PAH cations are adequately described by IR cooling alone in the SHC framework.^38^ However, recent experiments and calculations on known and likely cyano-PAHs,^39,40^ including 2CNI^+^,^41^ indicate substantial anharmonic couplings and departures from IR spectra computed using harmonic oscillators. The obvious question is, how significant are anharmonic effects in IR radiative cooling of molecules in space? Efforts to consider the influence of anharmonic effects in the radiative emissions profiles (band frequencies) of PAHs have been discussed in a range of works,^42–49^ although the impact of anharmonicity on IR radiative cooling rates over appropriate astronomical time scales (milliseconds to seconds) has not been considered.

Here, we describe an anharmonic cascade framework for modeling IR cooling rates and emission spectra. We demonstrate the model with 2CNI (Figure 1), supported by gas-phase mid-IR and cyrogenic condensed-phase electronic spectra. The choice of 2CNI as a clear demonstration is for four reasons: (i) 2CNI has been observed in TMC-1;^36^ (ii) we measure a gas-phase mid-IR absorption spectrum, which shows substantial anharmonic couplings and is consistent with anharmonic electronic structure calculations at the B3LYP/N07D level of theory, including resonance polyad matrices; a model is only as good as the quality of input parameters/parametrization; (iii) the expected contribution from recurrent fluorescence cooling for internal energies below the dissociation threshold is minimal, meaning that IR emission is the only active radiative cooling process; and (iv) we have performed cryogenic ion storage ring experiments at DESIREE on related cations to measure radiative cooling rates and find good accordance with master equation modeling. The performance of a new theoretical strategy involving the revDSDPBEP86/jun-cc-pVTZ method to describe the CN-stretch mode is discussed.

**Figure 1:**
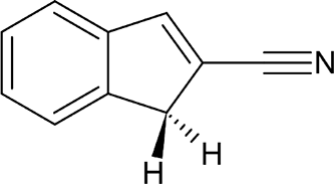
Illustration of the 2-cyanoindene molecule, which has *C*_*s*_ symmetry in the equilibrium geometry.

## 2 Methods

### 2.1 IR Spectroscopy

#### 2.1.1 Experimental Section

Gas phase mid-IR absorption spectra of 2CNI evaporated at room temperature were recorded at the AILES beamline situated at the SOLEIL synchrotron in France using a Bruker IFS125 Fourier transform Michelson-type interferometer, a multipass White-type cell, and a globar source.^50,51^ No synchrotron radiation was required for these experiments as a globar source of FIR continuum was adequate. The sample was leaked into the cell to achieve a static pressure of 2.5 μbar. The cell and interferometer were separated by ZnSe windows, and detection utilized a KBr beam splitter and a mercury cadmium telluride (MCT) detector. An initial spectrum was recorded with a resolution of 0.5 cm^–1^ and 1000 scans of each of transmission and reference over the 650–3200 cm^–1^ range. A second spectrum was recorded with a resolution of 0.05 cm^–1^, consisting of 200 scans of each of signal and reference over the 690–1650 cm^–1^ range. No unexpected or unusually high safety hazards were encountered.

#### 2.1.2 Theoretical

The anharmonic IR absorption spectrum for 2CNI was computed via a quartic force field (QFF), which is a truncated fourth-order Taylor expansion of the potential portion of the Watson Hamiltonian, given by

1 $$V=\frac{1}{2}\overset{3N}{\underset{i,j}{\sum }}\left(\frac{\partial^{2}V}{\partial X_{i}\partial X_{j}}\right)X_{i}X_{j}+\frac{1}{6}\overset{3N}{\underset{i,j,k}{\sum }}\left(\frac{\partial^{3}V}{\partial X_{i}\partial X_{j}\partial X_{k}}\right)X_{i}X_{j}X_{k}+\frac{1}{24}\overset{3N}{\underset{i,j,k,l}{\sum }}\left(\frac{\partial^{4}V}{\partial X_{i}\partial X_{j}\partial X_{k}\partial X_{l}}\right)X_{i}X_{j}X_{k}X_{l}$$

In the first step, the optimized geometries, normal modes, and harmonic frequencies were computed using the B3LYP^52^/N07D^53^ and rev-DSDPBEP86 ^54,55^ /jun-cc-pVTZ^56,57^ levels of theory with Gaussian 16.^58^ These computations were performed using very tight optimization criteria (1 × 10^–12^) and a custom integration grid consisting of 200 radial shells and 974 angular points per shell. The N07D basis set is based on the 6-31G basis set with additional dispersion and polarization functions that are known to improve the accuracy of vibrational frequency calculations of large aromatic molecules.^59^ The double-hybrid rev-DSDPBEP86 method combines density functional theory with second-order Møller–Plesset (MP2) electron correlation including Grimme’s D3BJ dispersion correction.^60^

In the second step, the normal coordinate QFF (quadratic, cubic, and semi-diagonal quartic force constants) was computed using small displacements (0.01 Å steps) along all normal coordinates, with a linear transformation providing a Cartesian coordinate QFF.^61^ Next, the vibrational spectrum was computed using second order vibrational perturbation theory (VPT2)^62–65^ within the software program Spectro.^66^ The VPT2 method implemented in Spectro utilizes a resonance polyad matrix approach.^67–69^ When two vibrational states of the same symmetry are close in frequency, they create a near-singularity in the conventional VPT2 equation. In the present approach, the interacting states were removed from the VPT2 computation and were included in resonance polyad matrices based on symmetry. These matrices allow for the treatment of resonance effects while also accounting for states that simultaneously participate in multiple resonance interactions, termed resonance chaining. Additionally, the resonance polyads treat the redistribution of intensity between coupled states by using the eigenvectors of the diagonalized matrix.^69,70^ The maximum frequency separation for a resonance in the polyad treatment is set to 200 cm^–1^.^69,71^ Vibrational modes with frequencies below 300 cm^–1^ are excluded from the VPT2 treatment due to known issues in the accurate description of their potential energy surfaces.^39,72–75^

Two different VPT2 approaches were used to compute the anharmonic IR absorption spectrum of 2CNI. The first approach utilized a standard anharmonic computation where the quadratic, cubic, and semidiagonal quartic force constants from the B3LYP/N07D level of theory were used in the VPT2 treatment. The second approach utilized a hybrid QFF, using the quadratic force constants from the rev-DSDPBEP86/jun-cc-pVTZ (abbreviated rDSD-TZ) computations and the cubic and semidiagonal quartic force constants from the B3LYP/N07D computations. Additionally, the intensities from the B3LYP/N07D calculation were used in the hybrid VPT2 treatment as they have better agreement with experiment. The hybrid method is abbreviated as rDSD-TZ+B3LYP. The original development and preliminary benchmarking of the rDSD-TZ+B3LYP hybrid method has been described in ref 76. The B3LYP/N07D method has been applied previously to PAHs with side groups^77–82^ and was recently applied to the cryogenic IR spectrum of 2CNI^+^,^41^ producing a mean absolute error (MAE) between theory and experiment of 4.3 cm^–1^ (excluding the CN-stretch mode). The MAEs for 2CNI^+^ frequencies from a range of other common density functional theory methods were substantially larger.

### 2.2 Electronic Fluorescence Spectroscopy

Electronic fluorescence excitation and emission spectra of 2CNI in methylcyclohexane (MeCH, Sigma-Aldrich, ≥99%), 2-methyltetrahydrofuran (2MeTHF, Acros, 99+%), and ethanol (EtOH, Fisher, 99.8%) were recorded at *T* = 77 K (samples in a 4 mm diameter EPR quartz tube immersed in a liquid nitrogen bath) using an Edinburgh Instruments FS5 spectrofluorometer. Spectra were acquired using an excitation bandwidth of 1.0 nm, an emission bandwidth of 0.5 nm, and a 0.2 s dwell time. The spectra in MeCH were recorded in 0.5 nm steps, and those in 2MeTHF and EtOH in 1 nm steps. The fluorescence excitation spectrum for molecules frozen in a glassy matrix at *T* = 77 K should parallel the absorption spectrum at the same temperature.^83^ No unexpected or unusually high safety hazards were encountered.

### 2.3 Radiative Cooling Model

The IR emission rate and emission spectrum of 2CNI was simulated using a master equation framework building on the SHC model for IR de-excitation that was developed to study the radiative cooling dynamics of vibrationally excited molecules.^33^ This strategy was developed against, and compared extensively with, ion storage ring experiments where the cooling of ensembles of ions with wide distributions of internal energy were tracked for tens of seconds using action spectroscopy methods,^34,84,85^ or by monitoring the yield of statistical processes competing with radiative cooling such as unimolecular dissociation and thermionic emission.^28–30,38,86^ The central goal in this work was not to derive highly accurate IR emission spectra, rather to incorporate anharmonic emission spectroscopy in a proven model that can also account for competing processes such as dissociation, isomerization, and recurrent fluorescence. Our treatment of anharmonic couplings, as described below, is thus simplified compared to the method described in section 2.1.1 for describing the IR absorption spectrum.

The IR emission simulation uses the anharmonic fundamental transition wavenumbers *h*ν_*a*_^1,0^, where *a* is the mode index, calculated at the B3LYP/N07D VPT2 theory. In addition, we include 1 + 1 combination band and first overtone transitions (wavenumbers and intensities), which are the standard output of a Gaussian 16 calculation using VPT2 theory. The vibrational modes are treated as separable Morse oscillators with vibrational energy levels

2 $$E_{a}^{n}=\omega_{e,a}\left(n+\frac{1}{2}\right)+\omega_{e}x_{e,a}{\left(n+\frac{1}{2}\right)}^{2}$$

where ω_*e*,*a*_ = *h*ν_*a*_^1,0^ – 2ω_*e*_ *x*_*e*,*a*_ and ω_*e*_ *x*_*e*,*a*_ is the diagonal element of the anharmonic **X** matrix. The vibrational density of states ρ­(*E*) is computed using the Stein-Rabinovitch extension^87^ of the Beyer–Swinehart algorithm.^88^

The emission rate coefficient for fundamental mode emission from level *n* to *n* – 1 of mode *a*, i.e., for the ν_*a*_^*n*^ → ν_*a*_^*n*–1^ transition, is given by^89^

3 $$k_{a}^{n,n-1}\left(E\right)=nA_{a}^{1,0}{\left(\frac{h\nu_{a}^{n,n-1}}{h\nu_{a}^{1,0}}\right)}^{3}\frac{\rho_{a}\left(E-E_{a}^{n}\right)}{\rho \left(E\right)}$$

where *E* is the total vibrational energy, *Eh*ν_*a*_^*n*,*n*–1^ = *E*_*a*_^*n*^ – *E*_*a*_^*n*–1^ is the transition wavenumber, *A*_*a*_^1,0^ is the Einstein coefficient of the ν_*a*_^1^ → ν_*a*_^0^ transition, and ρ_*a*_(*E*) is the density of states computed with the vibrational mode *a* excluded. Note the distinction between the transition wavenumbers *h*ν_*a*_^*n*,*m*^, the vibrational energy configurations ν_*a*_^*n*^, and the level energies *E*_*a*_^*n*^. The leading factor of *n* in eq 3 is the scaling factor for the Einstein coefficient in the harmonic oscillator, where the *n* → *n* – 1 transition is *n* times faster than the *n* = 1 → 0 transition. The factor ${\left(\frac{h\nu_{a}^{n,n-1}}{h\nu_{a}^{1,0}}\right)}^{3}$ is an approximate correction to this harmonic scaling which accounts for the cubic dependence of the Einstein coefficient on the transition energy.^89^ Finally, the ratio of level densities gives the probability that the vibrational mode *a* is occupied exactly *n* times. The total fundamental mode emission rate for the mode *a* is given by *k*_*a*_^fund^(*E*) = ∑_*n*_ *k*_*a*_^*n*,*n*–1^(*E*). The power radiated by this mode is *P*_*a*_^fund^(*E*) = ∑_*n*_ *h*ν_*a*_^*n*,*n*–1^ *k*_*a*_^*n*,*n*–1^(*E*). The total rate coefficient *k*_tot_^fund^ and radiative power *P*_tot_^fund^ for fundamental mode emission is found by summing over all vibrational modes *a*.

The rate coefficient for the first overtone ν_*a*_^*n*^ → ν_*a*_^*n*–2^ transition is given by^89^

4 $$k_{a}^{n,n-2}\left(E\right)=\frac{n\left(n-1\right)}{2}A_{a}^{2,0}{\left(\frac{h\nu_{a}^{n,n-2}}{h\nu_{a}^{2,0}}\right)}^{3}\frac{\rho_{a}\left(E-E_{a}^{n}\right)}{\rho \left(E\right)}$$

where *h*ν_*a*_^*n*, *n*–2^ = *E*_*a*_^*n*^ – *E*_*a*_^*n*–2^ is the transition wavenumber and *A*_*a*_^2,0^ is the Einstein coefficient of the ν_*a*_^2^ → ν_*a*_^0^ transition.

While there are many possible 1 + 1 combination bands, most have very low intensity. To reduce computational burden, only the combination bands with IR intensities greater than 1 km mol^–1^ are considered. The rate coefficient for 1 + 1 combination band emission in which mode *a* relaxes from level *n* to *n* – 1, and mode *b* from *m* to *m* – 1 is given by^89^

$$k_{a,b}^{n,n-1,m,m-1}\left(E\right)=nmA_{a,b}^{1,0,1,0}{\left(\frac{h\nu_{a,b}^{n,n-1,m,m-1}}{h\nu_{a,b}^{1,0,1,0}}\right)}^{3}\times \;\frac{\rho_{a,b}\left(E-E_{a,b}^{n,m}\right)}{\rho \left(E\right)}$$ 5

where *E*_*a*,*b*_^*n*,*m*^ = *E*_*a*_^*n*^ + *E*_*b*_^*m*^, *h*ν_*a*,*b*_^*n*,*n*–1,*m*,*m*–1^ = *E*_*a*,*b*_^*n*,*m*^ – *E*_*a*,*b*_^*n*–1,*m*–1^, *A*_*a*,*b*_^1,0,1,0^ is the Einstein coefficient of the ν_*a*_^1^ν_*b*_^1^ → ν_*a*_^0^ν_*b*_^0^ transition, and ρ_*a*,*b*_(*E*) is the density of states computed with both modes *a* and *b* excluded.

The IR emission rate coefficients are used to construct a population propagation matrix **U**. For example, each fundamental emission rate coefficient *k*_*a*_^*n*,*n*–1^(*E* – *h*ν_*a*_^*n*,*n*–1^) is added to the *h*ν_*a*_^*n*,*n*–1^th upper diagonal of **U**. The negative of the sum of all IR rate coefficients lies along the main diagonal such that ∑**U** = 0.

Energized 2CNI molecules may dissociate rather than radiatively cool if the internal vibrational energy exceeds the dissociation threshold. Our model includes destruction of 2CNI by hydrogen atom dissociation by incorporating the inverse Laplace transform model of the dissociation rate coefficient^90^

6 $$k^{\mathrm{diss}}\left(E\right)=A^{\mathrm{diss}}\frac{\rho \left(E-E_{a}\right)}{\rho \left(E\right)}$$

where the frequency factor *A* ^diss^ = 1.3 × 10^15^ s^–1^ was determined for the equivalent reaction in indene,^91^ which is similar (but slightly lower) to values for other gas-phase PAH cations.^92^ *E*_a_ = 3.51 eV is the dissociation asymptote calculated at the CCSD­(T)/cc-pVTZ level of theory including zero-point energy corrections using Gaussian 16.^56,93^ Because this is a simple bond scission, it is a barrierless (asymptotic) dissociation. No other dissociation channels were included because their calculated asymptotic dissociation energies were substantially higher (by >0.5 eV), and thus were not accessible over the internal vibrational energy range considered in this work.

#### 2.3.1 Internal Energy Distribution

IR emission properties, notably the emission spectrum, depend on the initial vibrational energy distribution, *g*(*E*,*t* = 0). For a physically meaningful simulation, *g*(*E*,*t* = 0) was initialized as the product of the measured cryogenic fluorescence excitation spectrum, which is a proxy for the ultraviolet–visible (UV–vis) absorption spectrum, and spectral energy density function that reflects the interstellar radiation field. For the latter, we used a blackbody spectrum at *T_BB_* = 6000 K as the energy density function to approximate light from a nearby F or G type star.^94^ The emission model assumes that the illuminated flux is sufficiently low such that any excited molecules cool completely before absorbing a second photon, i.e., the low-irradiation limit. In each step of the IR simulation, the energy distribution evolves according to the master equation.

7 $$g\left(E,t+dt\right)=\left[g\left(E,t\right)+Ug\left(E,t\right)dt\right]e^{-k^{\mathrm{diss}}\left(E\right)dt}$$

The wavenumber-resolved photon emission rate *I*(*h*ν,*t*) is calculated at each step for all transitions. For example, the rate of fundamental photon emission for the ν_*a*_^*n*^ → ν_*a*_^*n*–1^ is given by *I*(*h*ν_*a*_^*n*,*n*–1^, *t*) = ∫*k*_*a*_^*n*, *n*–1^(*E*)*g*(*E*,*t*)­d*E*. The total, time-integrated emission spectrum is *I*(*h*ν) = ∫*I*(*h*ν,*t*)­d*t*. Our IR cooling model is implemented in the so-called rapid exchange limit,^95^ where the rate of intramolecular vibrational energy redistribution is rapid compared with IR emission.^33^

## 3 Results and Discussion

### 3.1 IR Spectroscopy

The mid-IR spectrum recorded for 2CNI at *T* = 300 K is shown in Figure 2a, along with the B3LYP/N07D (Figure 2b) and rDSD-TZ+B3LYP (Figure 2c) calculated spectra. Peak frequencies from experiment, calculated frequencies, intensities (*I*) and assignments are given in Table 1 (see the Supporting Information for rDSD-TZ+B3LYP values). The mean absolute error (MAE) between theory and experiment is 6.5/4.1 cm^–1^ (including/excluding the CN stretch) for the B3LYP/N07D method (Table 1), and 6.8/6.5 cm^–1^ for the rDSD-TZ+B3LYP method. For comparison, the MAE for 2CNI^+^ with the B3LYP/N07D methodology was 4.3 cm^–1^ excluding the CN stretch mode.^41^ A summary of the fundamental modes at the B3LYP/N07D level of theory is given in the Supporting Information, while complete details of each calculated vibration is given in the zip file included in the Supporting Information. Overall, there is reasonable agreement between the B3LYP/N07D calculation and experiment, although the experimental spectrum has more structure than predicted by theory over the 850–1050 cm^–1^ range.

**Figure 2:**
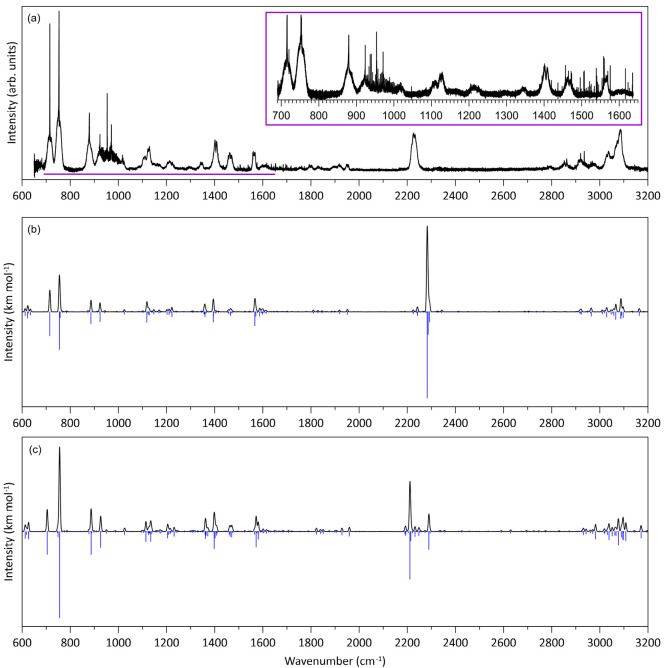
IR spectroscopy of gas-phase 2CNI: (a) mid-IR spectrum (0.5 cm^–1^ wavenumber increments) recorded at SOLEIL from vapor at *T* = 300 K. The inset shows the fingerprint region recorded in 0.05 cm^–1^ wavenumber increments. Sharp spikes present near the center of some of the IR bands are rotational Q-branches. (b) B3LYP/N07D anharmonic calculation at *T* = 0 K. (c) rDSD-TZ+B3LYP method anharmonic calculation at *T* = 0 K. In panels b and c, the black spectrum assumes the stick spectra (blue) convoluted with 5 cm^–1^ fwhm Gaussian functions.

**Table 1:** IR Spectra Properties of 2CNI^a^

expt.	calc.	*I*_calc._	assignments				% contributions			
715	715.0	22	ν_35_							
754	754.8	35	ν_34_							
879	886.0	11	ν_29_							
923	923.8	9	ν_28_							
1017	1024.4	2	ν_25_							
1114	1118.2	10	ν_23_	ν_29_ + ν_45_			57	33		
1219	1222.5	5	ν_18_							
1350	1359.6	5	ν_22_ + ν_45_	ν_26_ + ν_43_			67	18		
	1357.2	0.4	ν_18_ + ν_47_							
	1357.5	2	ν_33_ + ν_48_	ν_22_ + ν_45_	ν_15_		59	18	10	
	1359.6	5	ν_22_ + ν_45_	ν_26_ + ν_43_			67	18		
1401	1394.2	10	ν_14_	ν_26_ + ν_41_			66	23		
	1394.9	4	ν_26_ + ν_41_	ν_14_			65	25		
	1397.6	0.3	ν_25_ + ν_44_							
1464	1458.7	2	ν_13_	ν_28_ + ν_40_	ν_30_ + ν_38_		57	17	15	
	1466.0	3	ν_12_							
1563	1566.8	13	ν_11_	ν_32_ + ν_35_			72	17		
1800	1810.4	1	ν_27_ + ν_32_	ν_28_ + ν_29_			57	24		
1830	1829.0	1	ν_32_ + ν_36_							
1918	1917.9	2	ν_27_ + ν_26_							
1952	1951.3	2	2ν_26_							
2232	2282.8	80	ν_8_	ν_21_ + ν_23_			71	20		
	2286.8	21	ν_21_ + ν_23_	ν_20_ + ν_23_	ν_8_		55	29	14	
2920	2918.9	1	ν_7_	ν_9_ + ν_16_	ν_11_ + ν_15_		48	17	11	
	2922.9	2	ν_6_							
3037	3020.2	1	ν_11_ + ν_13_	ν_11_ + ν_12_			67	18		
	3028.5	5	ν_9_ + ν_13_	ν_5_	ν_3_		26	25	22	
	3046.6	2	ν_10_ + ν_12_	ν_11_ + ν_12_	ν_10_ + ν_13_	ν_4_	31	23	14	14
3066	3058.2	3	ν_4_	ν_3_			54	10		
	3066.4	7	ν_2_	ν_9_ + ν_12_	ν_4_	ν_8_ + ν_33_	37	28	12	10
	3066.7	1	ν_8_ + ν_33_							
3086	3086.9	6	ν_9_ + ν_12_	ν_2_	ν_9_ + ν_13_		40	25	12	
	3087.5	6	ν_10_ + ν_12_	ν_3_	ν_2_	ν_5_	38	18	14	11
	3089.9	3	ν_1_							
	3096.9	5	ν_9_ + ν_13_	ν_3_	ν_5_	ν_9_ + ν_12_	47	27	12	10

a Band center (cm^–1^), anharmonic frequency (cm^–1^), intensity (*I*_calc._, km mol^–1^), mode assignments, and percent contribution of the assigned modes from the B3LYP/N07D calculations. Uncertainties in experimental values are ±2 cm^–1^, limited by the breadth of the measured peaks rather than the radiation wavenumber.

The most significant feature in the IR spectrum for 2CNI is the CN-stretch mode measured at 2236 cm^–1^ (4.472 μm), which has a similar wavenumber to that for gas-phase benzonitrile at ≈2230 cm^–1^ reported in the NIST IR spectral database. The B3LYP/N07D calculation (*T* = 0 K with no rotational envelope) predicts the CN-stretch mode to be the most intense spectral feature, situated at 2283 cm^–1^, which is 47 cm^–1^ higher that experiment. This overestimation by theory is in common with 2CNI^+^ (at 37 cm^–1^).^41^ The rDSD-TZ+B3LYP method gave the CN-stretch frequency at 2211 cm^–1^, which is 21 cm^–1^ lower than the experimental value. On the other hand, the rDSD-TZ+B3LYP method was able to describe the CN stretch mode of 9-cyanoanthracene and two isomers of cyanonaphthalene isomers within 1 cm^–1^,^76^ suggesting that the underestimation for 2CNI may be due to the adjacent sp^2^ hybridization. Ultimately, further IR spectra for neutral and cationic cyano-PAHs are needed to fairly evaluate the rDSD-TZ+B3LYP method.

### 3.2 Fluorescence Spectroscopy

Electronic fluorescence excitation and emission spectra for 2CNI recorded at *T* = 77 K embedded in methylcyclohexane (MeCH), 2-methyltetrahydrofuran (2MeTHF), and ethanol (EtOH) glassy matrices are shown in Figure 3. 2CNI was not soluble in non-polar solvents such as alkanes. Peak spectral wavelengths from these spectra are given in Table 2. The MeCH fluorescence excitation spectrum has the most resolved structure and is considered as the best proxy to the absorption spectrum (excluding the Raman scattering peak) because MeCH is the least polar of the three solvents based on dielectric constants, ε, summarized in Table 2. In a first approximation, the 0–0 transition energy can be approximated as the average of absorption and emission maxima, at ≈303 nm (≈4.09 eV) in MeCH. Because RF must be preceded by inverse internal conversion,^35^ the 0–0 transition energy provides the lower-bound internal energy for RF.

**Figure 3:**
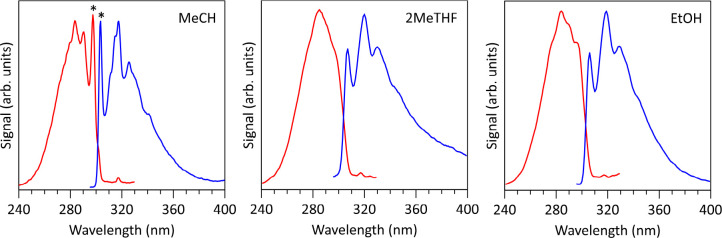
Fluorescence excitation (red) and emission (blue) spectra of 2CNI at *T* = 77 K frozen in glassy matrices of: methylcyclohexane (MeCH), 2-methyltetrahydrofuran (2MeTHF), and ethanol (EtOH). The excitation spectra were monitored emission at 350 nm (1 nm bandwidth), while the emission spectra excited at 285 nm (0.5 nm bandwidth). The feature denoted by ∗ in the MeCH spectra is an artifact from Raman scattering.

**Table 2:** Fluorescence Excitation/Absorption (abs) and Emission (ems) Wavelengths in nm for 2CNI Frozen in Glassy Matrices^a^

solvent	λ_abs_ (*T* = 300 K)	λ_abs_ (*T* = 77 K)^a^ ^,^ ^b^	λ_ems_ (*T* = 77 K)^b^
MeCH (ε = 2.02)^c^	279	283, 290	317, 326
2MeTHF (ε = 6.97)^c^	275	284	307, 320, 330
EtOH (ε = 25.3)^c^	278	284	306, 319, 329

a Uncertainties are ±1 nm.

b For MeCH, two λ_abs_ (*T* = 77 K) values are associated with vibronic structure.

c The two or three values correspond to vibronic structure.

d Dielectric constants, ε,^96^ at *T* = 293 K (298 K for 2MeTHF) indicate that MeCH is least polar and should have closest correspondence with the gas-phase environment.

### 3.3 IR Emission from 2CNI

#### 3.3.1 Emission Rates

Calculated IR emission rate coefficients from the anharmonic cascade model for fundamental, 1 + 1 combination band, first overtones, and total IR emission from 2CNI are shown in Figure 4 (top). The dissociation rate coefficient curve crosses the total IR emission rate coefficient curve at the critical energy *E*_c_ = 4.79 eV. Thus, most 2CNI molecules that absorb a photon of energy greater than *E*_c_ are destroyed and do not contribute to our simulated IR emission spectrum. It is worth noting that, while recurrent fluorescence is a dominant radiative cooling process for PAH cations with low-lying electronic excited states (e.g., 1–2.5 eV), the experimental band maximum for 2CNI at 4.38 eV (283 nm) from Table 2 means that recurrent fluorescence does not play a major role in radiative cooling and is thus excluded from the model–this is one of the reasons why 2CNI was selected as the first demonstration.

**Figure 4:**
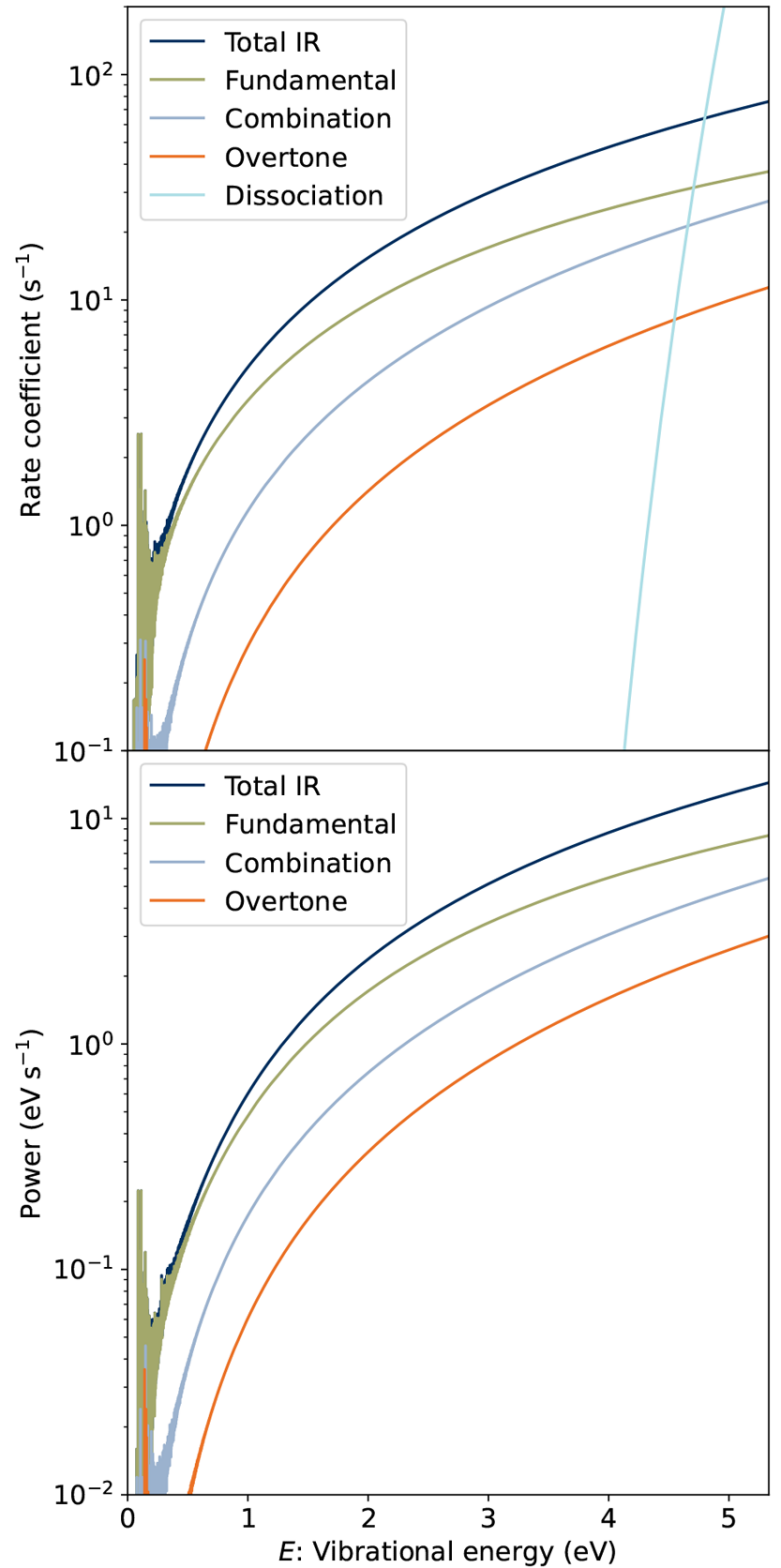
Calculated rates and IR-radiated power for 2CNI: (upper) Rate coefficients for IR emission and dissociation, with the former including fundamental (green), 1 + 1 combination (blue), and first overtone (orange) modes. The calculated dissociation limit is 3.51 eV. For internal energies >4.79 eV, dissociation dominates over IR cooling. (lower) IR-radiated power with total vibrational energy. While cooling is always dominated by the fundamental modes, 1 + 1 combination and overtone modes account for nearly half of the radiated power for internal energies near the dissociation threshold.

In addition to the emission rates, the IR-radiated power with vibrational energy, is shown in Figure 4 (bottom). For higher vibrational energies (e.g., 4 eV or more), emissions from 1 + 1 combination and first overtone bands account for about half the radiated power. For lower total vibrational energies (e.g., 1 eV and lower), emissions tend toward a 90:10:0 (fundamental:combination:overtone) ratio. These data clearly show that emission from anharmonic modes makes a significant contribution to the overall IR emission rate when 2CNI (and presumably other, similar cyano-PAHs) are energized with an amount of energy equivalent to a typical UV–vis photon (2.5–5 eV).

#### 3.3.2 Emission Spectra

The simulated IR emission spectrum, *I*(ν), from 2CNI is shown in Figure 5. This simulated spectrum assumes illumination by a *T*_BB_ = 6000 K blackbody spectrum as a proxy for a cool F or G type star near which IR emission from neutral PAHs might be expected, or a protoplanetary nebula like the Red Rectangle.^97^ The simulation was stopped at *t* = 10 s, at which time the total vibrational energy of the ensemble, *E*_tot_(*t*) = ∫*Eg*(*E*,*t*)­d*E*, was reduced by more than 98%. Due to the narrow window of energies that can be absorbed by a 2CNI molecule without leading to dissociation, the assumed blackbody temperature, *T*_BB_, in the simulation does not significantly alter the shape of *g*(*E*,0) or the resulting *I*(ν) (Figure 6). However, the irradiation temperature affects the fraction of molecules that undergo dissociation, ionization, or both, but this is beyond the scope of the present demonstration as we are interested in IR emission for internal energies that do not result in dissociation.

**Figure 5:**
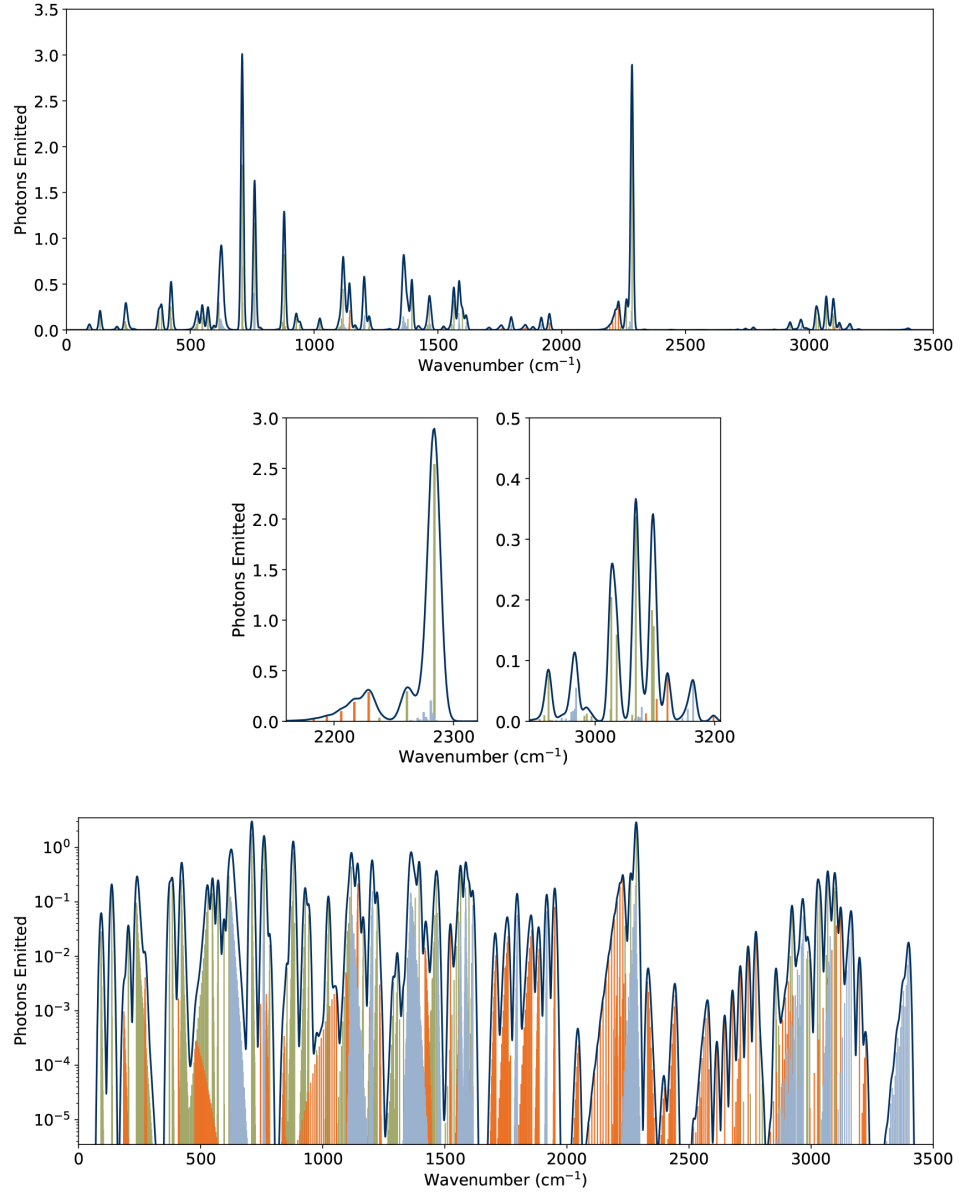
Simulated IR emission spectrum, *I*(ν), of 2CNI irradiated by a *T*_BB_ = 6000 K blackbody spectrum: (upper) linear vertical abscissa, and (lower) logarithmic vertical abscissa. (middle) Simulated IR emission spectrum for 2CNI over the CN-stretch (left) and CH-stretch (right) regions. Note that the vertical and horizontal axes are different in each panel. Stick spectra are calculated transition wavenumbers for fundamental (green), 1 + 1 combination (blue), and overtone (orange) bands. The continuous spectrum was determined by convolution of each stick with a Gaussian function with fwhm = 5 cm^–1^. The CN-stretch mode makes a substantial contribution to the emission spectrum, but is reduced in intensity compared with the IR absorption spectrum (Figure 2).

**Figure 6:**
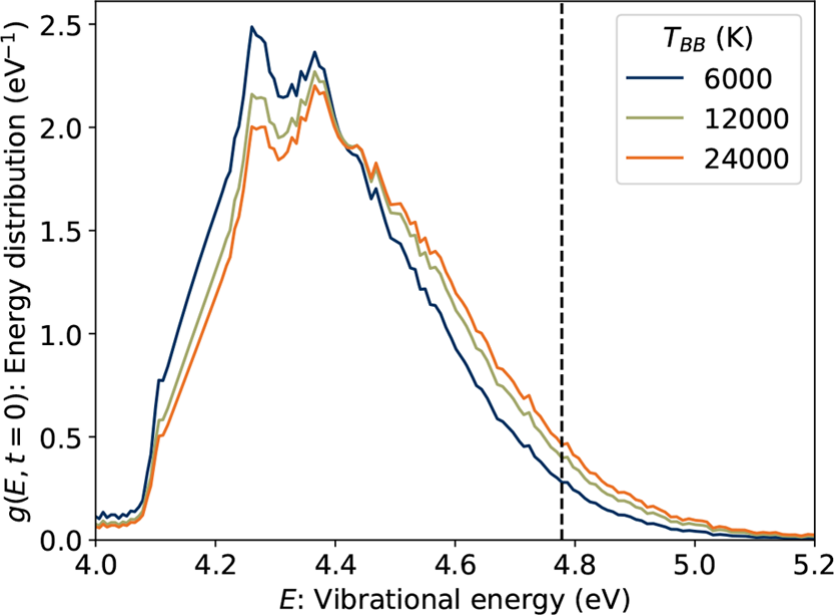
Initial vibrational energy distributions, *g*(*E*,0), in the master equation IR emission simulation for 2CNI, determined as the product of the fluorescence excitation (absorption) spectrum, and a blackbody spectrum with different temperature parameters, *T*_BB_. The dashed vertical line indicated the critical energy for dissociation in competition with radiative cooling, *E*_c_ = 4.79 eV. Note that this energy is somewhat higher than the dissociation limit at 3.51 eV.

Magnified portions of the CN and CH stretching regions of the simulated IR emission spectrum are shown in Figure 5. The CN-stretch region is dominated by the ν_8_^1^ → ν_8_^0^ transition calculated at 2285 cm^–1^ (4.377 μm). The ν_8_^2^ → ν_8_^1^ transition is clearly visible at 2262 cm^–1^. The progression between 2200–2240 cm^–1^ is due to the strong ν_23_^2^ → ν_23_^0^ overtone. The strongest 1 + 1 combination band also lies in this region: ν_23_^1^ν_21_^1^ → ν_23_^0^ν_21_^0^ at 2282 cm^–1^, but is buried under the ν_8_^1^ → ν_8_^0^ CN-stretch mode. The magnified CH stretching region in Figure 5 (middle) shows that the aromatic CH emission bands (ν_1_ through ν_5_) occur between 3000–3100 cm^–1^ (3.33–3.23 μm). The strongest aliphatic CH transition is ν_6_^1^ → ν_6_^0^ at 2923 cm^–1^ (3.42 μm). The ν_11_^2^ → ν_11_^0^ overtone is present at 3130 cm^–1^. Significant 1 + 1 combination band emission is seen from ν_15_^1^ν_9_^1^ → ν_15_^0^ν_9_^0^ at 2965 cm^–1^ and ν_11_^1^ν_10_^1^ → ν_11_^0^ν_10_^0^ at 3164 cm^–1^.

To illustrate the total contributions from anharmonic couplings, we have integrated the simulated total number of emitted photons and total energy dissipated per mode category (Figure 7). While the fundamental modes account for around two-thirds of the emission in both cases, the first overtone and 1 + 1 combination bands make a nearly one-third contribution. Ultimately, we conclude that overtone and combination modes make a substantial contribution to the IR emission spectrum for 2CNI.

**Figure 7:**
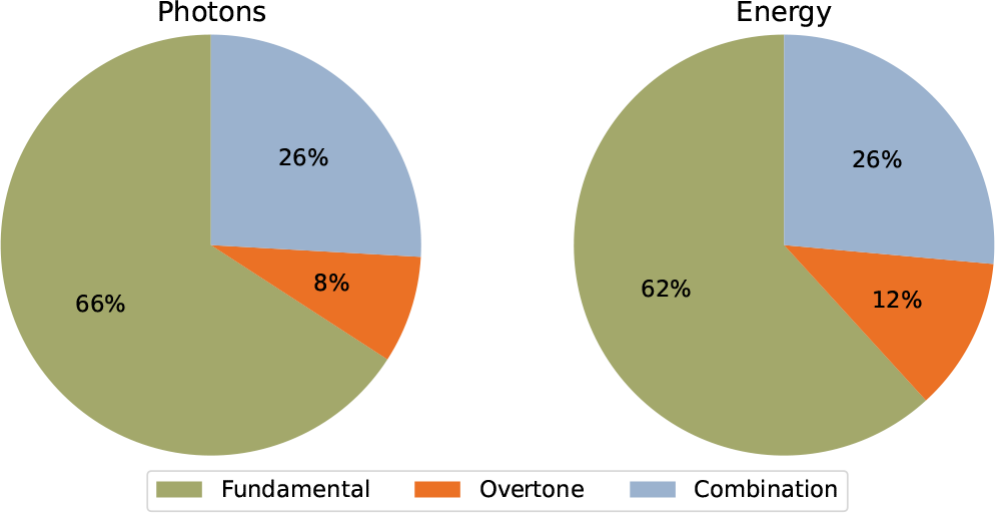
Total number of photons emitted (left) and total energy dissipated (right) by mode category for the 2CNI emission simulation presented in Figure 5. Emission from anharmoinc couplings (first overtone and 1 + 1 combination bands) contribute more than one-third of the number of emitted photons or total dissipated energy.

Comparison of the IR emission spectrum in Figure 5 with the (calculated) IR absorption spectrum in Figure 2 clearly shows suppression of the CN-stretch mode, which is not a pronounced feature in observed AIBs, relative to modes in the fingerprint region. Notably, the calculated ν_35_ = 724 cm^–1^ (symmetric out-of-plane CH wag) and ν_34_ = 767 cm^–1^ (symmetric out-of-plane CH bend) modes are pronounced with the latter having the same intensity as the CN-stretch mode. In simple terms, the lower frequency modes become enhanced in IR emission, compared with IR absorption spectra, for high total vibrational energies (i.e., after absorption of an UV–vis photon) because they have an increasing relative probability to radiate during de-excitation (cooling) of the internal energy distribution with concurrent intramolecular vibrational energy redistribution.^11^ The ν_35_ and ν_34_ modes are of particular interest because they are close to the asymmetric 12.7 μm AIB observed by astronomers,^98,99^ which is attributed to out-of-plane C–H bending modes in neutral PAHs.^100^ Future developments to our model could include temperature-dependent anharmonic frequency effects to recover asymmetries on the emission band shapes to allow for a direct comparison between simulated emission spectra and astronomical observations.

## 4 Conclusion

We have reported an anharmonic cascade model in a master equation framework to simulate IR emission rates and emission spectra of energized PAH molecules present in space. The model builds on the simple harmonic cascade model, also implemented in a master equation framework, which was developed in conjunction with ion storage ring experiments probing the radiative cooling dynamics and rates of isolated carbonaceous ions. In contrast to other anharmonic IR emission models in the literature, which are focused on temperature-dependent IR emission band properties, our model has been developed from the perspective of emission rates to allow for comparison with “ultraslow” ion storage ring experiments performed under background conditions similar to cold, dark molecular clouds. Our example application to 2-cyanoindene, which is one of the few specific PAHs that has been discovered in TMC-1, shows substantial contributions from anharmonic couplings to both the IR emission rate and spectra, clearly demonstrating that anharmonicity needs to be considered in reliable models of IR emission rates.

Because the output of a model is only as good as the quality of the input data or training parameters, we recorded a mid-IR absorption spectrum for 2CNI and compared with state-of-the-art electronic structure calculations of anharmonic IR absorption spectra. The B3LYP/N07D level of theory including resonance polyad matrices gave overall reasonable agreement, although the CN-stretch mode frequency continues to be difficult to describe. Consequently, we can be confident that the emission spectrum should be reasonable. In a similar vein, because the underlying SHC model is able to reproduce total IR emission rates within a factor of 2 or so of experiments for a range of carbonaceous ions, including PAH cations, we have confidence that the IR emission rates should be reliable.

Our anharmonic cascade model is easily incorporated into a greater master equation relaxation dynamics model framework,^28,29,31^ which may include competing processes such as recurrent fluorescence, multiple dissociation or isomerization pathways. The interplay between these pathways usually needs be considered for larger PAHs, particularly if the molecule is energized through absorption of a VUV or XUV photon that raises the internal energy above dissociation threshold(s). While it is difficult to make any universal statements about the importance of anharmonicity in IR spectra across PAHs as a class, because the total (integrated) intensity in a calculated IR absorption or emission spectrum typically increases when anharmonic effects are included, models incorporating robust anharmonic effects should give enhanced IR emission rates. Our current example of 2CNI presented an ideal illustration for IR-emission-dominated radiative cooling because the contribution from recurrent fluorescence is expected to be minimal. On the other hand, the combination of IR emission and recurrent fluorescence becomes increasing important for larger PAHs as electronic states typically become systematically lower in energy with increasing size.^28,29,31,36,37^ To further assess the influence of anharmonicity in small astro-PAHs, future work by our collaboration will seek to compare modeled IR emission rates with measured radiative cooling rates for a series of PAH cations that offer differing extents of anharmonic couplings.

## Supplementary Material





## Notes

Calculated vibrational frequencies and assignments using the rDSD-TZ+B3LYP method, summary of harmonic modes, comparison of computed anharmonic and harmonic IR spectra, and simulated IR emission spectra with delta-function energies (PDF)
Complete details of each calculated vibration (ZIP)
