# Supplementary material for: Infrared Cooling in an Anharmonic Cascade Framework: 2‑Cyanoindene, the Smallest Cyano-PAH Identified in Taurus Molecular Cloud‑1
Source: ACS Earth Space Chem. 2025 Feb 4;9(2):382–93. doi: 10.1021/acsearthspacechem.4c00381 (PMC11850017; doi:10.1021/acsearthspacechem.4c00381)
Supplement: Supplementary file 1 [file sp4c00381_si_001.pdf]

# Supporting Information for 'Infrared Cooling in an Anharmonic Cascade Framework: 2-Cyanoindene, the Smallest Cyano-PAH Identified in TMC-1'

Mark H. Stockett,[†] Vincent J. Esposito,[‡] Eleanor K. Ashworth,[¶] Ugo Jacovella,[§] and James N. Bull[*,¶]

†*Department of Physics, Stockholm University, SE-10691 Stockholm, Sweden*

‡*NASA Ames Research Center, Moffett Field, CA 94035, USA*

¶*Chemistry, Faculty of Science, University of East Anglia, Norwich NR4 7TJ, United Kingdom*

§*Institut des Sciences Moléculaires d'Orsay, CNRS, Université Paris-Saclay, F-91405 Orsay, France*

E-mail: james.bull@uea.ac.uk

Table S1: IR spectral properties of 2CNI. Calculated band center ($cm^{-1}$), anharmonic frequency ($cm^{-1}$), intensity ($I_{B3LYP}$, km $mol^{-1}$), mode assignments, and percent contribution of the assigned modes from the rDSD-TZ+B3LYP method. Uncertainties in experimental values are $\pm 2\,cm^{-1}$, limited by the breadth of the measured peaks rather than the radiation wavenumber.

| Expt | Hybrid | $I$ | Assignments | | | | % contributions | | | |
|---|---|---|---|---|---|---|---|---|---|---|
| 715 | 704.1 | 12 | $\nu_{35}$ | | | | | | | |
| 754 | 755.7 | 46 | $\nu_{34}$ | | | | | | | |
| 879 | 886.5 | 12 | $\nu_{30}$ | | | | | | | |
| 923 | 926.2 | 8 | $\nu_{28}$ | | | | | | | |
| 1017 | 1025.6 | 2 | $\nu_{25}$ | $2\nu_{40}$ | | | | | | |
| 1114 | 1114.1 | 5 | $\nu_{23}$ | $2\nu_{38}$ | $\nu_{30}+\nu_{45}$ | | 43 | 28 | 20 | |
| 1219 | 1231.5 | 2 | $\nu_{18}$ | | | | | | | |
| 1350 | 1360.2 | 1 | $\nu_{26}+\nu_{43}$ | $\nu_{15}$ | $\nu_{27}+\nu_{42}$ | | 66 | 12 | 10 | |
| | 1361.2 | 4 | $\nu_{22}+\nu_{45}$ | $\nu_{26}+\nu_{43}$ | $\nu_{18}+\nu_{47}$ | | 52 | 20 | 19 | |
| | 1362.2 | 1 | $\nu_{18}+\nu_{47}$ | $\nu_{22}+\nu_{45}$ | | | 79 | 19 | | |
| | 1364.0 | 2 | $\nu_{27}+\nu_{42}$ | $\nu_{15}$ | $\nu_{22}+\nu_{45}$ | $\nu_{26}+\nu_{43}$ | 46 | 21 | 17 | 10 |
| 1401 | 1397.8 | 9 | $\nu_{14}$ | $\nu_{30}+\nu_{40}$ | | | 59 | 33 | | |
| | 1401.4 | 3 | $\nu_{30}+\nu_{40}$ | $\nu_{32}+\nu_{39}$ | $\nu_{14}$ | | 48 | 22 | 21 | |
| 1464 | 1460.3 | 1 | $\nu_{34}+\nu_{35}$ | | | | | | | |
| | 1464.0 | 3 | $\nu_{13}$ | $\nu_{29}+\nu_{39}$ | $\nu_{34}+\nu_{35}$ | $\nu_{12}$ | 44 | 16 | 15 | 14 |
| 1563 | 1565.3 | 1 | $\nu_{32}+\nu_{35}$ | | | | | | | |
| 1800 | 1791.6 | 0.1 | $\nu_{20}+\nu_{36}$ | | | | | | | |
| 1830 | 1822.7 | 1 | $\nu_{27}+\nu_{32}$ | $\nu_{26}+\nu_{34}$ | | | 82 | 12 | | |
| 1918 | 1928.6 | 2 | $\nu_{27}+\nu_{26}$ | | | | | | | |
| 1952 | 1959.3 | 2 | $2\nu_{26}$ | | | | | | | |
| 2232 | 2211.0 | 25 | $\nu_{8}$ | $2\nu_{24}$ | | | 64 | 13 | | |
| | 2214.6 | 5 | $2\nu_{24}$ | $\nu_{8}$ | | | 83 | 13 | | |

| *Expt* | *Hybrid* | *I* | *Assignments* | | | | | *% contributions* | | | | |
|---|---|---|---|---|---|---|---|---|---|---|---|---|
| | 2232.0 | 3 | $\nu_{23}+\nu_{24}$ | | | | | | | | | |
| 2920 | 2923.8 | 0.2 | $\nu_9+\nu_{17}$ | $\nu_9+\nu_{16}$ | | | | 81 | 13 | | | |
| | 2927.1 | 0.2 | $2\nu_{13}$ | $2\nu_{12}$ | | | | 59 | 14 | | | |
| | 2931.4 | 2 | $\nu_7$ | $2\nu_{13}$ | | | | 68 | 11 | | | |
| 3037 | 3018.0 | 1 | $\nu_5$ | $\nu_{11}+\nu_{13}$ | | | | 44 | 26 | | | |
| | 3032.1 | 2 | $\nu_{11}+\nu_{12}$ | $\nu_{11}+\nu_{13}$ | | | | 54 | 25 | | | |
| | 3037.9 | 4 | $\nu_{11}+\nu_{13}$ | $\nu_3$ | $\nu_5$ | | | 26 | 22 | 17 | | |
| | 3051.3 | 2 | $\nu_{10}+\nu_{13}$ | $\nu_{11}+\nu_{12}$ | $\nu_{11}+\nu_{13}$ | $\nu_3$ | $\nu_{10}+\nu_{12}$ | 31 | 19 | 12 | 10 | 10 |
| 3066 | 3062.3 | 2 | $\nu_{10}+\nu_{12}$ | $\nu_{10}+\nu_{13}$ | | | | 38 | 28 | | | |
| | 3067.4 | 2 | $\nu_4$ | | | | | | | | | |
| | 3076.9 | 7 | $\nu_2$ | $\nu_9+\nu_{12}$ | | | | 45 | 17 | | | |
| 3086 | 3089.6 | 3 | $\nu_1$ | | | | | | | | | |
| | 3095.3 | 8 | $\nu_{10}+\nu_{12}$ | $\nu_5$ | $\nu_{10}+\nu_{13}$ | $\nu_3$ | | 32 | 18 | 17 | 17 | |
| | 3097.2 | 4 | $\nu_9+\nu_{13}$ | $\nu_9+\nu_{12}$ | $\nu_2$ | | | 38 | 28 | 26 | | |

Table S2: Assignments of the fundamental (harmonic) modes for 2CNI calculated at the B3LYP/N07D level of theory.

| Mode | $\nu_{harm}$ ($cm^{-1}$) | Description of motion |
|---|---|---|
| $\nu_1$ | 3229.694 | Aromatic CH stretch |
| $\nu_2$ | 3208.193 | Symmetric aromatic CH stretch |
| $\nu_3$ | 3197.360 | Asymmetric aromatic CH stretch |
| $\nu_4$ | 3187.902 | Asymmetric aromatic CH stretch |
| $\nu_5$ | 3181.470 | Asymmetric aromatic CH stretch |
| $\nu_6$ | 3074.631 | Asymmetric aliphatic CH stretch |
| $\nu_7$ | 3042.965 | Symmetric aliphatic CH stretch |
| $\nu_8$ | 2314.298 | CN stretch |
| $\nu_9$ | 1653.898 | CC skeletal stretch |
| $\nu_{10}$ | 1635.433 | CC skeletal stretch |
| $\nu_{11}$ | 1602.062 | CC skeletal stretch |
| $\nu_{12}$ | 1494.461 | CC skeletal stretch |
| $\nu_{13}$ | 1490.657 | CC skeletal stretch |
| $\nu_{14}$ | 1434.580 | Aliphatic CH scissor |
| $\nu_{15}$ | 1388.117 | CC skeletal stretch |
| $\nu_{16}$ | 1331.032 | Aromatic CH in-plane wag |
| $\nu_{17}$ | 1321.566 | CC skeletal stretch |
| $\nu_{18}$ | 1246.568 | CC skeletal stretch |
| $\nu_{19}$ | 1227.502 | Symmetric CH wag |
| $\nu_{20}$ | 1188.793 | Symmetric CH wag |
| $\nu_{21}$ | 1179.317 | Asymmetric in-plane aromatic CH bend |
| $\nu_{22}$ | 1147.694 | Asymmetric aliphatic CH torsion |
| $\nu_{23}$ | 1143.761 | Symmetric in-plane CH bend |
| $\nu_{24}$ | 1119.941 | Asymmetric in-plane CH bend |

| *Mode* | $\nu_{harm}$ *(cm$^{-1}$)* | *Description of motion* |
|---|---|---|
| $\nu_{25}$ | 1041.898 | Asymmetric in-plane CH bend |
| $\nu_{26}$ | 993.299 | Asymmetric out-of-plane CH bend |
| $\nu_{27}$ | 961.717 | Asymmetric out-of-plane CH bend |
| $\nu_{28}$ | 944.190 | Asymmetric out-of-plane CH bend |
| $\nu_{29}$ | 905.099 | Asymmetric out-of-plane CH bend |
| $\nu_{30}$ | 898.817 | In-plane CC skeletal breathing |
| $\nu_{31}$ | 883.555 | In-plane CC skeletal breathing |
| $\nu_{32}$ | 871.719 | Asymmetric out-of-plane CH wag |
| $\nu_{33}$ | 795.932 | In-plane CC skeletal breathing |
| $\nu_{34}$ | 766.881 | Symmetric out-of-plane CH bend |
| $\nu_{35}$ | 724.254 | Symmetric out-of-plane CH wag |
| $\nu_{36}$ | 629.073 | In-pane CC skeletal deformation |
| $\nu_{37}$ | 603.326 | In-pane CC skeletal deformation |
| $\nu_{38}$ | 576.483 | In-plane CCN bend |
| $\nu_{39}$ | 556.662 | Out-of-plane skeletal torsion |
| $\nu_{40}$ | 539.976 | Out-of-plane CCN bend |
| $\nu_{41}$ | 427.838 | Out-of-plane skeletal torsion |
| $\nu_{42}$ | 427.707 | In-plane skeletal elongation |
| $\nu_{43}$ | 387.864 | Out-of-plane aromatic CH rock |
| $\nu_{44}$ | 375.393 | Asymmetric In-plane rock |
| $\nu_{45}$ | 240.631 | Out-of-plane CC skeletal wag |
| $\nu_{46}$ | 206.931 | Out-of-plane aliphatic CH wag |
| $\nu_{47}$ | 136.445 | In-plane CN wag |
| $\nu_{48}$ | 90.353 | Out-of-plane CN wag |

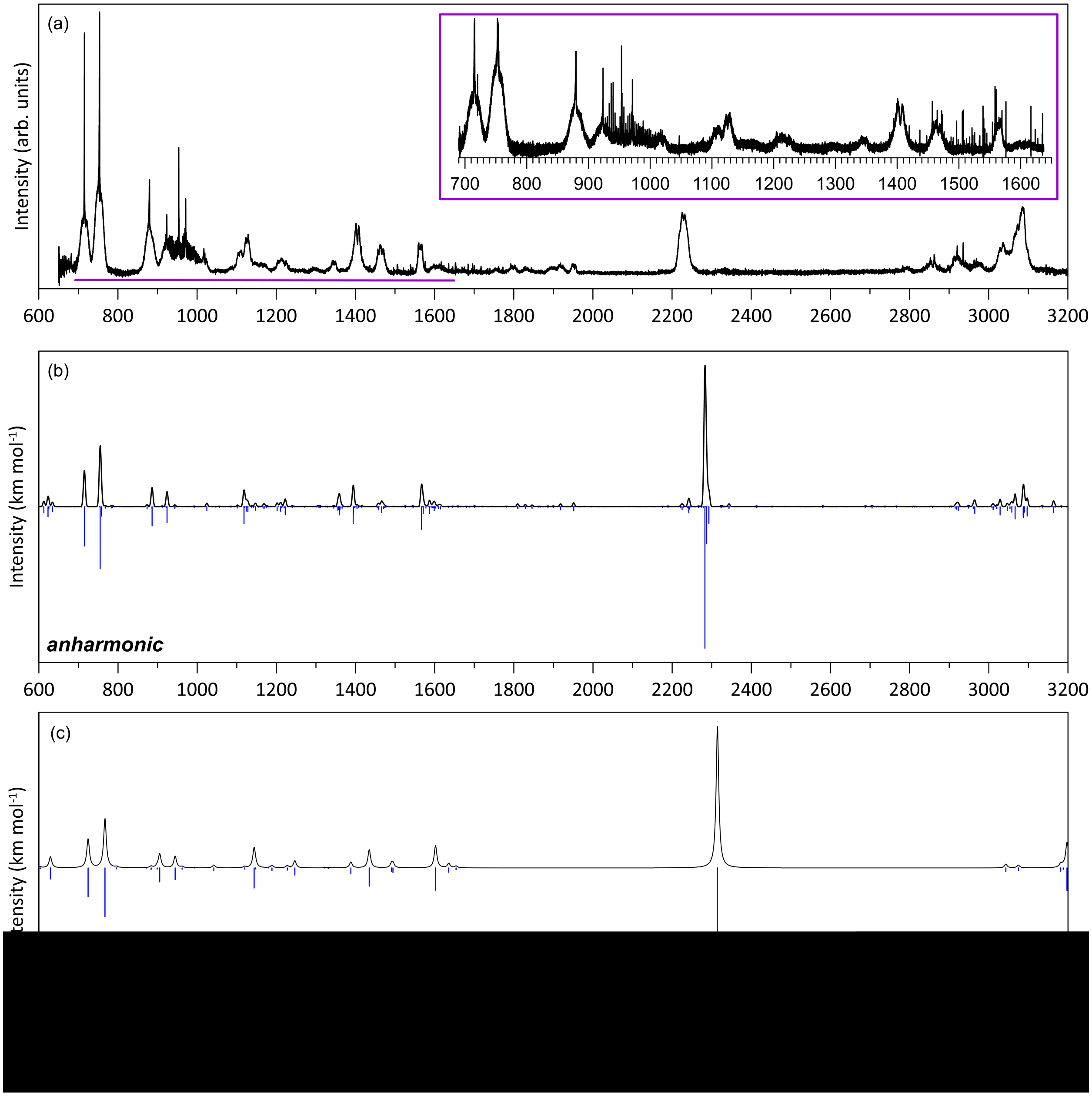


[redacted]

Figure S1: 2CNI IR spectra: (a) experimental spectrum, (b) B3LYP/N07D anharmonic calculation at $T = 0\,\mathrm{K}$. Both panels (a) and (b) are reproduced from Figure 2 in the paper. (c) B3LYP/N07D harmonic calculation at $T = 0\,\mathrm{K}$. Note that the harmonic calculation is unable to describe the C-H stretching region over 2800–3200 $\mathrm{cm}^{-1}$.

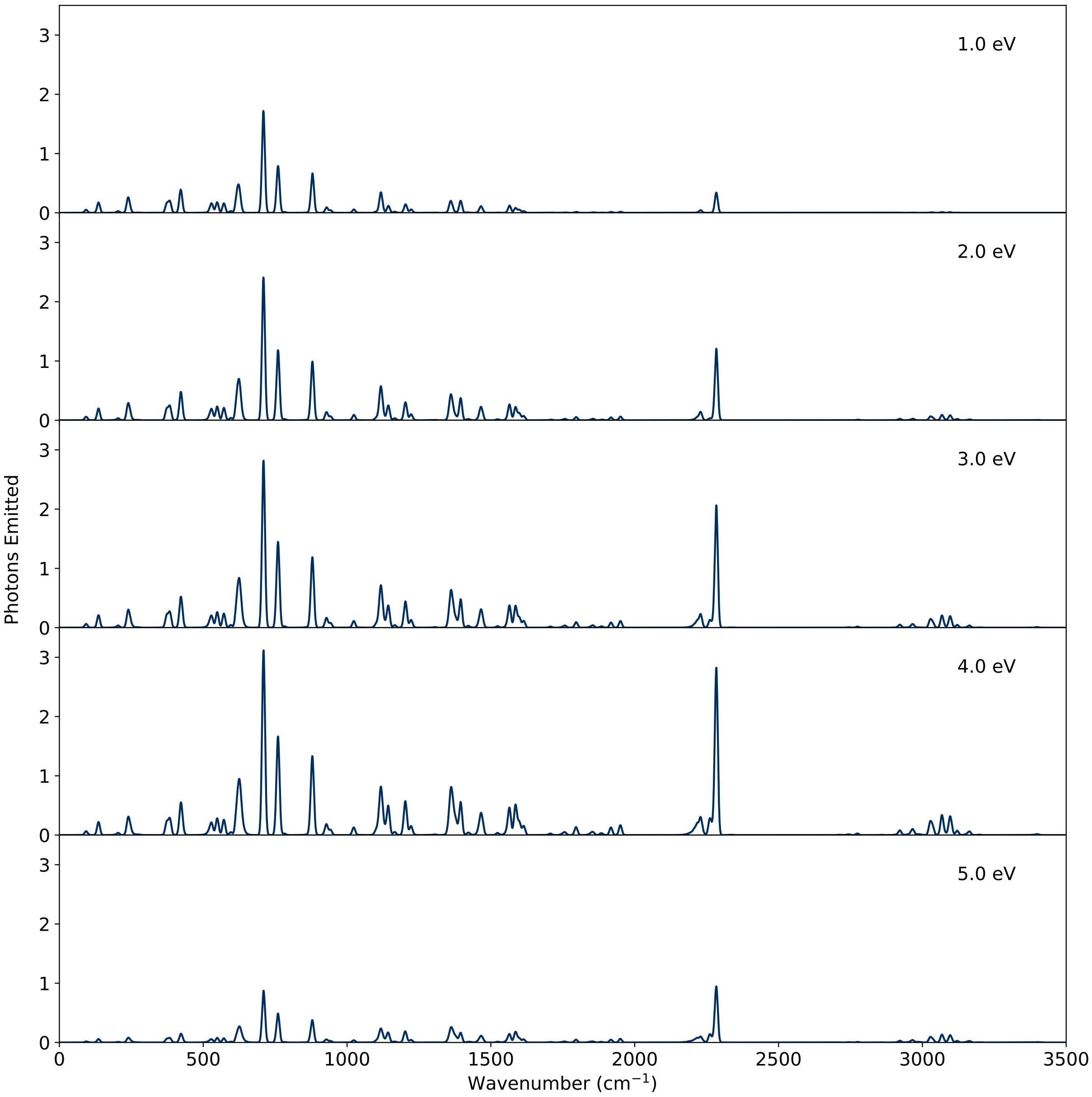


Figure S2: Simulated IR emission spectrum, $I(\nu)$, of 2CNI with $g(E, 0)$ initialized to delta functions. The CN-stretch and CH-stretch modes are suppressed for low internal energies (1–2 eV), and most pronounced at 4 eV. Above 4 eV, dissociation becomes the dominant pathway and the IR emission spectrum becomes weak. Suppression of the CN-stretch mode at low internal energies means that it may not be as useful as indicated by IR absorption spectra as a spectroscopic marker for cyano-PAHs in astronomical AIB observations.
